# In silico prediction of *Gallibacterium anatis* pan-immunogens

**DOI:** 10.1186/s13567-014-0080-0

**Published:** 2014-08-08

**Authors:** Ragnhild J Bager, Egle Kudirkiene, Isabelle da Piedade, Torsten Seemann, Tine K Nielsen, Susanne E Pors, Andreas H Mattsson, John D Boyce, Ben Adler, Anders M Bojesen

**Affiliations:** Department of Veterinary Disease Biology, Faculty of Health and Medical Sciences, University of Copenhagen, 1870 Frederiksberg C, Denmark; Victorian Bioinformatics Consortium, Monash University, 3800 Clayton, Melbourne, Australia; The Novo Nordisk Foundation Center for Protein Research, Faculty of Health and Medical Sciences, University of Copenhagen, 2200 Copenhagen N, Denmark; Center for Biological Sequence Analysis, Technical University of Denmark, 2800 Lyngby, Denmark; Evaxion Biotech North America LLC, Wilmington, USA; Department of Microbiology, Monash University, 3800 Clayton, Melbourne Australia; Australian Research Council Centre of Excellence in Structural and Functional Microbial Genomics, Department of Microbiology, Monash University, 3800 Clayton, Melbourne Australia

## Abstract

The Gram-negative bacterium *Gallibacterium anatis* is a major cause of salpingitis and peritonitis in commercial egg-layers, leading to reduced egg production and increased mortality. Unfortunately, widespread multidrug resistance and antigenic diversity makes it difficult to control infections and novel prevention strategies are urgently needed. In this study, a pan-genomic reverse vaccinology (RV) approach was used to identify potential vaccine candidates. Firstly, the genomes of 10 selected *Gallibacterium* strains were analyzed and proteins selected on the following criteria; predicted surface-exposure or secretion, none or one transmembrane helix (TMH), and presence in six or more of the 10 genomes. In total, 42 proteins were selected. The genes encoding 27 of these proteins were successfully cloned in *Escherichia coli* and the proteins expressed and purified. To reduce the number of vaccine candidates for in vivo testing, each of the purified recombinant proteins was screened by ELISA for their ability to elicit a significant serological response with serum from chickens that had been infected with *G. anatis*. Additionally, an in silico prediction of the protective potential was carried out based on a protein property prediction method. Of the 27 proteins, two novel putative immunogens were identified; Gab_1309 and Gab_2312. Moreover, three previously characterized virulence factors; GtxA, FlfA and Gab_2156, were identified. Thus, by combining the pan-genomic RV approach with subsequent in vitro and in silico screening, we have narrowed down the pan-proteome of *G. anatis* to five vaccine candidates. Importantly, preliminary immunization trials indicated an in vivo protective potential of GtxA-N, FlfA and Gab_1309.

**Electronic supplementary material:**

The online version of this article (doi:10.1186/s13567-014-0080-0) contains supplementary material, which is available to authorized users.

## Introduction

Poultry meat and eggs are considered very important and sustainable sources of animal protein worldwide [1], making efficient strategies to prevent and control the spread of poultry diseases highly important [2]. *Gallibacterium anatis* is a Gram-negative, non-motile, encapsulated coccobacillus of the *Pasteurellaceae* family [3,4] and commonly associated with poultry [5]. Besides constituting a part of the normal microflora of the upper respiratory tract and lower genital tract in chickens [6], it is also considered a major cause of salpingitis and peritonitis in egg-laying chickens [7-9]. Thus, *G. anatis* infections lead to a drop in egg production and increased mortality in commercial layers [10]. Unfortunately, widespread multiple-drug resistance [11] hinders treatment with traditional antimicrobial agents, while substantial antigenic diversity [12] among disease-causing field isolates hampers disease prevention by classical vaccination with inactivated whole cell bacterins. Hence, novel prevention strategies are urgently needed.

The sequencing of the first bacterial genome in 1995 [13] initiated the genomic era and catalyzed a shift from conventional culture-based approaches to genome-based vaccinology [14]. This gave rise to the Reverse Vaccinology (RV) approach [15], in which bioinformatics tools are used to analyze genome sequences to identify genes encoding likely protective antigens. The concept of RV was initially applied to *Neisseria meningitidis* serogroup B (MenB) [16], for which conventional vaccine development approaches had failed in producing an efficacious vaccine. Based on the genomic sequence of MenB strain MC58 [17], five universal vaccine candidates were identified [18], and the resulting 4CMenB vaccine (Bexsero®) is now approved in the EU for active immunization of individuals aged over two months against disease caused by MenB [19]. Since this pioneering MenB project, the RV approach has been applied to a variety of other important pathogens [20]. However, the increased availability of multiple genomes for the same bacterial species has shown that genomic variability in bacteria is much more extensive than initially anticipated. Thus, analysis of the genome of a single strain often fails to address intra-species genetic variability and limits the effectiveness of genome-wide screens for vaccine candidates. To overcome this, a pan-genomic RV model utilizing the global gene repertoire for a species was proposed by Tettelin et al. [21]. Pan-genomic RV was first applied to vaccine development in Group B *Streptococcus* [22], and this study demonstrated the importance of sequencing multiple strains of a single pathogen for the identification of vaccine antigens [23]. The application of in silico and in vitro predictions has not only enabled a much more rational selection of vaccine candidates, but has also shown promise at reducing the number of experimental animals needed to verify the effectiveness of vaccine candidates.

We report here the use of a pan-genomic RV approach for identification of novel and conserved immunogens of *G. anatis*. By implementing different in silico approaches and in vitro assays, we screened the *Gallibacterium* pan-proteome, resulting in a final selection of five proteins with a high predicted potential as vaccine candidates. Importantly, preliminary in vitro immunization results indicate protective potential of at least three of these candidates including FlfA, which has previously been tested and confirmed highly protective against homologous challenge in chickens [24]. Together, these results provide an important step in the development of a new and broadly protective vaccine against *G. anatis*.

## Materials and methods

### Animal ethics statement

All work on experimental animals was carried out with the approval of the Danish National Animal Ethics Committee (Approval no. 2012-15-2934-00339 and 2012-15-2934-00923).

### *Gallibacterium* strains and growth conditions

The 10 *Gallibacterium* strains included in the study are listed in Table 1. The strains were selected based on their pathogenic potential, prevalence in the field and genetic diversity, in order to provide as much diversity as possible within the species. Bacteria were incubated at 37 °C on brain heart infusion (BHI) agar supplemented with 5% citrated bovine blood in a closed plastic bag or in BHI broth with aeration.

**Table 1:** ***Gallibacterium*** **strains for included in this study**

**Strain**	**Biovar**	**Host/tissue**	**Lesions**	**Country** ^**a**^	**Reference**
*G. anatis* bv. *anatis*					
Biovar *anatis*					
F149^T^	NA	Duck/intestine	-	DK	[3]
Biovar *haemolytica*					
12656-12	4	Chicken/liver	+	DK	[25]
10672-6	1	Chicken/oviduct	+	DK	[3]
4895	4	Chicken/NA	+	MX	[26]
7990	3	Chicken/NA	+	MX	[26]
Avicor	4	Chicken/heart	+	MX	[26]
CCM5995	20	Chicken/NA	NA	Cz	[3]
IPDH 697-78	15	Chicken/NA	+	G	[3]
*G.* genomospecies 1					
CCM5974	8	Hen/Liver	+	Cz	[3]
*G.* genomospecies 2					
CCM5976	9	Hen/oviduct	+	Cz	[3]

^a^Cz: Czech Republic, DK: Denmark, G: Germany, MX: Mexico.

^T^ = type strain.

NA = Not available.

### RV in silico analysis

Sequencing and assembly of *G. anatis* strains 12656–12 and F149^T^ was performed as described previously [27]. The remaining genomes were sequenced using the Illumina Genome Analyzer IIx (CD genomics, New York, USA). Reads were assembled using VelvetOptimiser 2.0 [28]. All 10 genomes were annotated using Prokka v.1.0 [29]. The subcellular localization of the 31 564 annotated proteins from the 10 genomes was predicted using standalone PSORTb v.3.0 [30]. The presence of N-terminal signal peptides was predicted using SignalP v.3.0 [31] and the number of transmembrane helices (TMHs) was predicted by TMHMM v.2.0 [32]. The protein conservation among the strains was analyzed using BLASTp v.2.2.22 [33] with default parameters. The theoretical molecular masses and isoelectric points were calculated using the pepstats tool in EMBOSS [34]. In total, 42 proteins were selected (Table 2).

**Table 2:** **The 42 proteins from** ***G. anatis*** **12656–12 selected for cloning and expression**

**Protein ID**	**Description**	**Mw (kDa)**	**PSORTb prediction**	**TMHMM prediction**	**SignalP prediction**	**Acc. no.**
Gab_0001	Hypothetical protein	195.9	Outer membrane	0	No	ERF78007.1
Gab_0047	Metal-dependent proteases with possible chaperone activity	36.5	Extracellular	0	No	ERF77629.1
Gab_0087	Hypothetical protein	140.9	Extracellular	0	No	ERF78219.1
Gab_0091	Outer membrane lipoprotein	15.3	Unknown	0	Yes	ERF79624.1
Gab_0151^a^	RTX toxins and related Ca2 + −binding proteins	216.4	Extracellular	0	No	FJ917356
Gab_0178	Predicted secreted acid phosphatase	30.5	Unknown	0	Yes	ERF78374.1
Gab_0186	Membrane-bound lytic murein transglycosylase	39.9	Unknown	0	Yes	ERF78366.1
Gab_0337	Autotransporter adhesin	142.3	Extracellular	0	No	ERF78595.1
Gab_0523	Outer membrane protein and related peptidoglycan-associated (lipo)proteins	16.4	Outer membrane	0	Yes	ERF78926.1
Gab_0572^b^	F17-like fimbrial subunit	19.1	Extracellular	0	Yes	ERF79277.1
Gab_0574	P pilus assembly protein, porin PapC	94.2	Outer membrane	0	Yes	ERF79276.1
Gab_0602	Outer membrane protein	47.8	Outer membrane	0	Yes	ERF78505.1
Gab_0652	Organic solvent tolerance protein OstA	90.1	Outer membrane	0	Yes	ERF78644.1
Gab_0661	Small protein A (tmRNA-binding)	16	Unknown	0	Yes	ERF78651.1
Gab_0925	Hypothetical protein	85.2	Outer membrane and/or extracellular	0	Yes	ERF77284.1
Gab_0999	Rare lipoprotein B	18.2	Unknown	1	Yes	ERF79357.1
Gab_1008	Type II secretory pathway, component HofQ	42.1	Outer membrane	0	No	ERF79423.1
Gab_1162	P pilus assembly protein, porin PapC	93.8	Outer membrane	0	Yes	ERF78846.1
Gab_1164^b^	F17-like fimbrial subunit	20.5	Extracellular	0	Yes	JX855927
Gab_1192	Surface lipoprotein	28	Cytoplasmic membrane	0	Yes	ERF78832.1
Gab_1245	Hemolysin activation/secretion protein	67.8	Outer membrane	1	Yes	ERF78979.1
Gab_1283	Long-chain fatty acid transport protein	48.8	Outer membrane	0	Yes	ERF77302.1
Gab_1309	Membrane proteins related to metalloendopeptidases	42.7	Unknown	0	Yes	ERF77527.1
Gab_1396	Uncharacterized protein conserved in bacteria	146.6	Outer membrane	1	No	ERF79175.1
Gab_1397	Outer membrane protein	65.9	Outer membrane	0	Yes	ERF79124.1
Gab_1399	Membrane-bound metallopeptidase	47.2	Outer membrane and/or extracellular	0	Yes	ERF79126.1
Gab_1450	Opacity protein and related surface antigens	23.1	Outer membrane	1	Yes	ERF79004.1
Gab_1576	Outer membrane receptor for ferrienterochelin and colicins	23.4	Outer membrane	0	No	ERF78509.1
Gab_1631	Cell envelope opacity-associated protein A	43.9	Extracellular	1	No	ERF79417.1
Gab_1654	Outer membrane phospholipase A	32.4	Outer membrane	1	Yes	ERF79322.1
Gab_1755	Outer membrane protein and related peptidoglycan-associated (lipo)proteins	27.9	Outer membrane	0	Yes	ERF79542.1
Gab_2087	Outer membrane protein	51	Outer membrane	0	Yes	ERF78059.1
Gab_2124^c^	Outer membrane protein (porin)	41.1	Outer membrane	0	Yes	KF160335
Gab_2156^b^	F17-like fimbrial subunit	20.7	Extracellular	0	Yes	ERF79559.1
Gab_2158	P pilus assembly protein, porin PapC	90.6	Outer membrane	0	Yes	ERF79560.1
Gab_2192	Outer membrane protein W	25.7	Outer membrane	0	Yes	ERF78317.1
Gab_2224	Outer membrane receptor proteins, mostly Fe transport	81	Outer membrane	0	Yes	ERF78217.1
Gab_2274	Outer membrane receptor proteins, mostly Fe transport	74	Outer membrane	0	Yes	ERF77464.1
Gab_2304	Glycerophosphoryl diester phosphodiesterase	41.6	Unknown	0	Yes	ERF77421.1
Gab_2312	Autotransporter adhesin	325.7	Outer membrane and/or extracellular	0	No	ERF77433.1
Gab_2347	Outer membrane protein/protective antigen OMA87	89.3	Outer membrane	0	Yes	ERF79042.1
Gab_2348	Outer membrane protein	19.8	Outer membrane	0	Yes	ERF79081.1

^a^Previously described as GtxA in [35].

^b^Previously described in [24]. Gab_1164 = FlfA.

^c^Previously described as OmpC in [36].

### Cloning and small-scale protein expression

Each of the selected genes was amplified from the *G. anatis* 12656–12 genome by PCR and cloned into the Gateway entry vector pENTR™/SD/D-TOPO (Invitrogen). Primers were designed using Oligo Explorer 1.2 (Gene Link™, Hawthorne, NY, USA) as described previously [37]. Areas with high predicted hydrophobicity in the N and/or C terminus were removed, as were predicted signal peptides. In addition, GtxA was cloned as two parts (N- and C-terminal) due to its size. The *E. coli* strains and plasmids used in this study are listed in Additional file 1. Genes were cloned and small-scale expressed as described in Additional file 2 using Gateway cloning and ligation-independent cloning (LIC) systems. Altogether, 37 expression clones were constructed for 36 of the 42 selected proteins (two clones were made for GtxA). The primer sequences used for gene amplification, and the final expression vector chosen for protein expression from each gene, are listed in Additional file 3.

### Large-scale protein expression and purification

All proteins were expressed and purified in large-scale from *E. coli* Rosetta 2 (DE3) cells (Novagen, Madison, WI, USA). Large-scale expression was performed in a custom-made large-scale expression system (LEX) (Harbinger Biotech, Toronto, Canada) as described previously [38] and in Additional file 2. Of the 37 expression clones, 27 recombinant proteins were successfully purified; the majority (17) of these proteins had a purity > 90%.

### Production of antiserum against *G. anatis* 12656–12 in chickens

Two Lohmann brown chickens (21 weeks old) were purchased from a commercial breeder with high biosecurity standards. The chickens were kept under free indoor housing conditions and were provided with fresh water and feed *ad libitum*. The chickens were swabbed for the presence of *G. anatis* by a cloacal swab. After two weeks of acclimatization the chickens were challenged with 10^5^ colony forming units (CFU) of *G. anatis* 12656–12 by injection into the peritoneal cavity as previously described [39] and re-infected 2 weeks after the first infection. Blood for serum purification was collected from the brachial vein prior to the first infection (pre-immune antiserum) and one week after the second infection (hyper-immune antiserum).

### Enzyme-Linked Immunosorbent Assay (ELISA)

The putative immunogenicity of each of the purified recombinant proteins was assessed by indirect ELISA as described previously [40], using pooled anti-*G. anatis* pre-immune and hyper-immune antiserum. Briefly, Nunc-Immuno™ MicroWell™ 96-Well Plates (Thermo Scientific, Waltham, MA, USA) were coated overnight at 4 °C with 0.5 μg recombinant protein (48 wells per protein) diluted in carbonate-bicarbonate buffer (pH 9.6) (Sigma-Aldrich, St. Louis, MO, USA). Each well was then washed; this and all subsequent washing steps consisted of three washes in 350 μL wash buffer (PBS + 0.05% Tween 20). The wells were blocked for 2 h at room temperature in 200 μL blocking solution (PBS containing 0.05% Tween 20 and 2% bovine serum albumin (BSA)) and washed. The antibody titers were assayed by serial 3-fold dilutions of chicken serum ranging from 1:200 to 1:48600. All dilutions were prepared in triplicate in dilution buffer (PBS containing 0.05% Tween 20 and 0.1% BSA), 100 μL were added to each well and plates were incubated for 1 h at 37 °C. For each assay, 12 control wells were included, which contained pure dilution buffer; secondary antibody was added to 6 of these wells as a measure of background, and the other 6 wells remained blank as a negative control for the ELISA. Following incubation, the wells were washed and 100 μL polyclonal goat anti-chicken IgG (Fc):HRP (AbD Serotec, Puchheim, Germany), diluted 1:4000 in diluting buffer, were added to each well and the plates incubated for a further 1 h at 37 °C and then washed. To detect the binding, 100 μL of 3,3′,5,5′-Tetramethylbenzidine (TMB) liquid substrate (Sigma) were added to each well. The plates were incubated for 2 min and then the reaction was stopped by addition of 100 μL 1 M HCl. The absorbance was read immediately at 450 nm in a PowerWave XS spectrophotometry (BioTek Instruments, Winooski, VT, USA).

The antibody titers were calculated for the measured absorbances at 450 nm [41], using the “Antibody Titers” online data analysis tool [42]. To compare and rank the ELISA results, a P/N ratio (P = hyper-immune serum, N = pre-immune serum) of mean antibody titers was calculated [43]. All statistical analysis was performed using SAS version 9.3 (SAS Institute, Cary, NC, USA), and differences between groups assessed using a one-way t-test. The recognition of recombinant protein by hyper-immune serum was deemed significant at *P* < 0.05, indicating that the protein was expressed in vivo during *G. anatis* infection and elicited a specific immune response.

### VacFinder® in silico protein analysis

To further predict the protective potential of each of the expressed proteins, each of the proteins was analyzed using the proprietary VacFinder® in silico technology platform (Evaxion Biotech, LLC, USA). VacFinder® is a data-driven machine learning method trained by protein property pattern recognition on known and protective B-cell protein antigens (except for exotoxins), aiming at identifying novel and protective B-cell protein antigens with a neutralizing opsonizing profile. The machine-learned prediction is based on specific protein property features of protein sequences rather than sequence similarity, allowing antigen classification based solely on protein properties [44]. The output is a list of proteins from the proteome ranked by their ability to elicit a highly protective antibody response.

### Immunization of layer chickens with recombinant proteins

24 Isa Brown layer chickens (16 weeks old) were purchased from a commercial breeder with high biosecurity standards. The chickens were swabbed for the presence of *G. anatis* by a cloacal swab. The chickens were randomly divided in eight groups of four each and allowed to acclimatize for one week after arrival. The chickens were kept under free indoor housing conditions and provided with fresh water and feed *ad libitum*. Each group was immunized subcutaneously with 100 μg of one of the five selected recombinant proteins (GtxA-N, FlfA, Gab_1309, Gab_2156 or Gab_2312) mixed in 0.5 mL of SEC buffer (50 mM NaP, 150 mM NaCl, 0.5 mM TCEP, 10% glycerol; pH 7.5) and 0.5 mL of Freund’s incomplete adjuvant (Sigma-Aldrich). As a control (non-immunized), a group of four chickens was immunized with a placebo (SEC buffer and Freund’s incomplete adjuvant). Two weeks after the immunization all chickens were infected intraperitoneally with 1.5 × 10^6^ CFU of *G. anatis* 12656–12 as described previously [39]. Forty-eight hours after infection the chickens were euthanized and a post mortem examination was conducted. To assess the protective effect of the immunization, the lesions found in peritoneum of each bird were scored according to three parameters: (i) inflammatory reaction, (ii) amount of exudate, and (iii) clarity of the peritoneal surfaces. All parameters were scored on a scale from 0–3, thus giving a maximum score of 9. Furthermore, the presence of *Gallibacterium* was detected by swabbing the peritoneum with a sterile cotton swab and streaking it onto BHI blood agar. The scorings of the lesions in the peritoneum were analyzed by a Mann Whitney U test and *P* < 0.05 were deemed significant.

### Multiple sequence alignments

Multiple amino acid sequence alignments of the Gab_1309 and Gab_2312 proteins and their orthologs were prepared using MAFFT v7.130b [45] and formatted using Jalview 2.8.0b1 [46].

### Genbank accession numbers

The genome sequence of *G. anatis* 12656–12 has recently been made available [47] and was submitted to Genbank (BioProject ID: 213810, accession number AVOX00000000). The nucleotide sequence accession numbers for the genes included in this study are listed in Table 2. Genome sequence reads from the nine *Gallibacterium* strains used for Gab 1309 and Gab_2312 multiple sequence alignment were submitted to the NCBI Sequence Read Archive (SRA) [48] and can be retrieved using the study accession number SRP029613.

## Results

### RV in silico prediction of candidate vaccine antigens

For the identification and selection of putative immunogens, the genomes of 10 *Gallibacterium* strains were analyzed (Table 1). Based on the central premise that protective antigens should be accessible to the host immune system, proteins predicted to be surface-exposed or secreted were selected from the *G. anatis* pan-proteome. Moreover, proteins with more than one TMH were discarded, based on the premise that they are unlikely to be transported beyond the inner membrane. In addition, these proteins have the highest rate of expression failure during subsequent procedures [16] or are less likely to be over-expressed in *E. coli* [49]. Finally, proteins present in six or more of the 10 *G. anatis* genomes were identified. A protein was considered present if a significant full length match (E-value < 10^−8^) was obtained. From a total of 31564 proteins, 162 proteins were predicted as extracellular proteins and 482 proteins as outer membrane proteins. Of these, 42 proteins were present in six or more of the 10 genomes and these proteins were selected for further studies (Table 2).

### Cloning, expression and purification

Each of the 42 selected genes was amplified from the *G. anatis* strain 12656–12 genome by PCR. This strain was chosen for gene cloning as it is a well-characterized and highly pathogenic strain originally isolated from the liver of a chicken with septicaemia [25,39]. Using different high-throughput cloning strategies, 37 proteins were successfully purified at a small-scale from *E. coli* and of these, 27 were successfully purified in the large-scale trials (Table 3). The 10 remaining proteins were lost during the large-scale purification process due to low yield, impurities or lack of expression in *E. coli* in large-scale cultures.

**Table 3:** **The 27 large-scale purified proteins**

**Protein ID**	**Description**	**Form**	**P/N ratio** ^**a**^	**Acc. no.**
GtxA-N^c^	RTX toxin (first half part)	Soluble	20.1***	FJ917356
Gab_0523	Outer membrane protein and related peptidoglycan-associated (lipo)proteins	Soluble	7.9***	ERF78926.1
Gab_2156^d^	F17-like fimbrial subunit	Soluble	6.6***	ERF79559.1
Gab_0091	Outer membrane lipoprotein	Insoluble	5.9***	ERF79624.1
Gab_2348	Outer membrane protein	Insoluble	4.1***	ERF79081.1
FlfA^d^	F17-like fimbrial subunit	Soluble	2.5***	JX855927
Gab_1755	Outer membrane protein and related peptidoglycan-associated (lipo)proteins	Insoluble	1.8***	ERF79542.1
Gab_1309	Membrane proteins related to metalloendopeptidases	Soluble	1.7***^b^	ERF77527.1
Gab_2312	Autotransporter adhesion	Soluble	1.7***^b^	ERF77433.1
GtxA-C^c^	RTX toxin (last half part)	Insoluble	1.4***	FJ917356
Gab_1576	Outer membrane receptor for ferrienterochelin and colicins	Insoluble	1.6*	ERF78509.1
Gab_1654	Outer membrane phospholipase A	Insoluble	1.3*	ERF79322.1
Gab_2224	Outer membrane receptor proteins, mostly Fe transport	Insoluble	1.3	ERF78217.1
Gab_2192	Outer membrane protein W	Insoluble	1.2	ERF78317.1
Gab_1450	Opacity protein and related surface antigens	Insoluble	1.2	ERF79004.1
Gab_0652	Organic solvent tolerance protein OstA	Insoluble	1.2	ERF78644.1
Gab_1283	Long-chain fatty acid transport protein	Insoluble	1.2	ERF77302.1
Gab_0047	Metal-dependent proteases with possible chaperone activity	Soluble	1.0	ERF77629.1
Gab_1162	P pilus assembly protein, porin PapC	Insoluble	1.0	ERF79423.1
Gab_1192	Surface lipoprotein	Insoluble	1.0	ERF78832.1
Gab_1397	Outer membrane protein	Insoluble	1.0	ERF79124.1
Gab_0925	Hypothetical protein	Insoluble	1.0	ERF77284.1
Gab_0186	Membrane-bound lytic murein transglycosylase	Insoluble	1.0	ERF78366.1
Gab_2304	Glycerophosphoryl diester phosphodiesterase	Soluble	0.9	ERF77421.1
OmpC^e^	Outer membrane protein (porin)	Insoluble	0.9	KF160335
Gab_0572^d^	F17-like fimbrial subunit	Insoluble	0.9	ERF79277.1
Gab_1245	Hemolysin activation/secretion protein	Insoluble	0.9	ERF78979.1

^a^Ratio between the mean antibody titer values (P = hyper-immune serum; N = pre-immune serum). Asterisks indicate statistically significant difference between the two groups (* = *P* < 0.05, *** *P* < 0.001).

^b^Proteins ranked within the Top20 of the *G. anatis* 12656–12 proteome by VacFinder®.

^c^Previously described in [35]. Due to the size of the protein it was cloned, expressed and purified as two halves.

^d^Previously described in [24].

^e^Previously described in [36].

### Screening of immunogenic potential by ELISA

**Figure 1:**
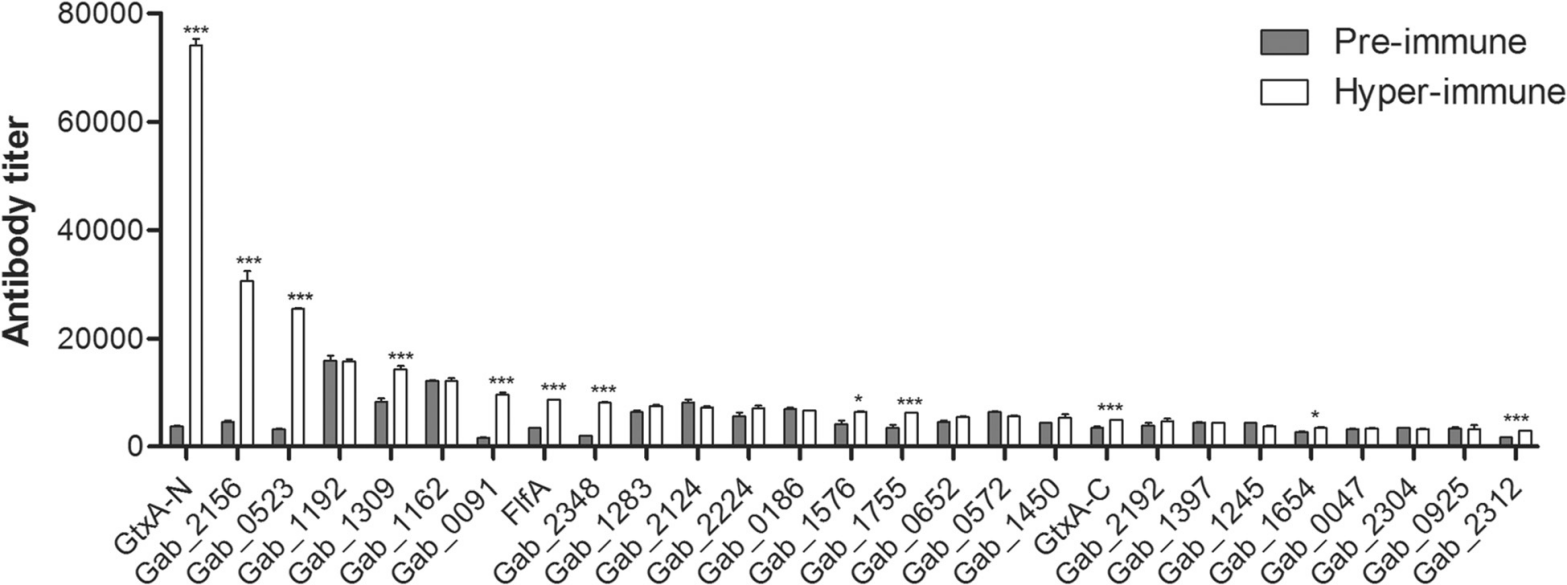
**Antibody titers of immune serum against recombinant proteins.** Mean calculated antibody titers ± SEM are shown for each protein based on the ELISA performed on sera collected before (pre-immune) and after (hyper-immune) infection with *G. anatis* strain 126565–12. The titers were calculated using the measured absorbances at 450 nm as described in the text. Differences in mean antibody titers between pre-immune and immune ELISA responses were analyzed using one-way ANOVA with a t test. Asterisks indicate statistical significance (*P < 0.05, ***P < 0.001).

In order to reduce the number of antigens to be tested in vaccine trials, the immunogenic potential of the 27 purified proteins was assessed in vitro by an indirect ELISA approach using pools of anti-*G. anatis* 12656–12 pre-immune and hyper-immune antiserum from two chickens. The chickens were, as expected, found to be positive for *G. anatis* on the cloacal mucosa prior to the immunizations. The mean antibody titers were calculated for each protein using both pre-immune and hyper-immune antiserum. To rank the proteins, P/N values between the two antibody titers were calculated for each protein (Table 3). A significant and specific reaction with hyper-immune antiserum was identified for 12 of the 27 recombinant proteins tested (Figure 1), namely GtxA (both halves of the protein), FlfA, Gab_0091, Gab_0523, Gab_1309, Gab_1575, Gab_1654, Gab_1755, Gab_2156, Gab_2312 and Gab_2348. These results indicate (i) that these 12 proteins are expressed in vivo by *G. anatis* 12656–12, (ii) that the proteins are recognized by the chicken immune system, and (iii) that the proteins elicit a specific antibody response. The remaining proteins were not significantly recognized by the hyper-immune antiserum.

### Screening of protective potential by VacFinder®

To provide an additional prediction of the protective potential of the 27 proteins available in recombinant form, the protein sequences were analyzed in silico with the VacFinder® algorithm. The entire *G. anatis* strain 12656–12 proteome (approximately 2500 proteins) was analyzed and ranked for protective potential. Proteins ranked in the Top20 are those predicted to have the highest protective potential (Table 3). Of the 27 proteins cloned and expressed in *E. coli*, only Gab_1309 and Gab_2312 were ranked within the Top20 VacFinder® hits.

### Conservation of Gab_1309 and Gab_2312

Two of the 27 proteins, Gab_1309 and Gab_2312, elicited a significant ELISA response and were ranked in the Top20 proteins with protective potential in the *G. anatis* 12656*–*12 proteome as determined by VacFinder®, suggesting that these proteins should be prioritized in further analyses. Both proteins were expressed in *E. coli* with an N-terminus His_6_-tag and purified in soluble form. Due to the high degree of predicted hydrophobicity within the C-terminal region of Gab_2312, as well as the size of the whole protein (325.7 kDa), only a small part (338 residues) was included in the final recombinant protein. Homologs of Gab_1309 and Gab_2312 were identified within the genomes of all the 10 strains included in this study, as well as in the recently sequenced *G. anatis* strain UMN179 (UMN_1211 and UMN_1565) [27], further supporting their potential as broadly protective immunogens. To determine the level of protein sequence conservation of Gab_1309 and Gab_2312, multiple alignments were conducted against the protein homologs from other *G. anatis* strains. The Gab_1309 protein sequences were almost identical across all strains (Additional file 4). On the other hand, the Gab_2312 protein sequences varied considerably in length from 2157 residues in *G. anatis* strain F149 to 5202 residues in *G.* genomospecies 2 strain CCM5976. Despite this difference, several well-conserved sections could be identified within the sequences, indicating a degree of conservation within the structure and the presence of common epitopes. Additional file 5 shows the alignment between the 338 residues included in the recombinant protein and the corresponding parts in the protein homologs.

### Potential protective capacity

**Figure 2:**
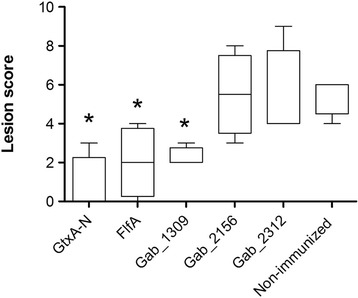
**Immunization of chickens with recombinant proteins.** Chickens were immunized once with 100 μg recombinant protein (GtxA-N, FlfA, Gab_1309, Gab_2156, or Gab_2312) or a placebo (non-immunized), followed by intraperitoneal challenge with *G. anatis* 12656–12. The scoring of the lesions found in the peritoneum was done according to three parameters: (i) inflammatory reaction, (ii) amount of exudate, and (iii) clarity of the peritoneal surfaces. All parameters were scored on a scale from 0–3, thus giving a maximum score of 9. The horizontal lines shows the group median, and the difference between the lesions scores of immunized and non-immunized groups was analyzed using Mann Whitney U test. Asterisks indicate statistical significance (*P < 0.05).

To evaluate the protective potential in vivo of the five most promising vaccine candidates (GtxA-N, FlfA, Gab_1309, Gab_2156, or Gab_2312), groups of four chickens were each immunized with one of the recombinant proteins, followed by intraperitoneal challenge with *G. anatis* 12656–12. *G. anatis* was recovered in pure culture from chickens within all the immunized groups, as well as from the non-immunized group. However, two chickens from the group immunized with GtxA-N and one chicken from the group immunized with FlfA were culture-negative. A significant lower lesion score was found in the group immunized with GtxA-N (*P* = 0.02), FlfA (*P* = 0.04) and Gab_1309 (*P* = 0.02) when compared to the non-immunized group (Figure 2), indicating a protective potential of these three proteins. The two groups immunized with Gab_2156 or Gab_2312 did not show a significant difference when compared with the non-immunized group.

## Discussion

**Figure 3:**
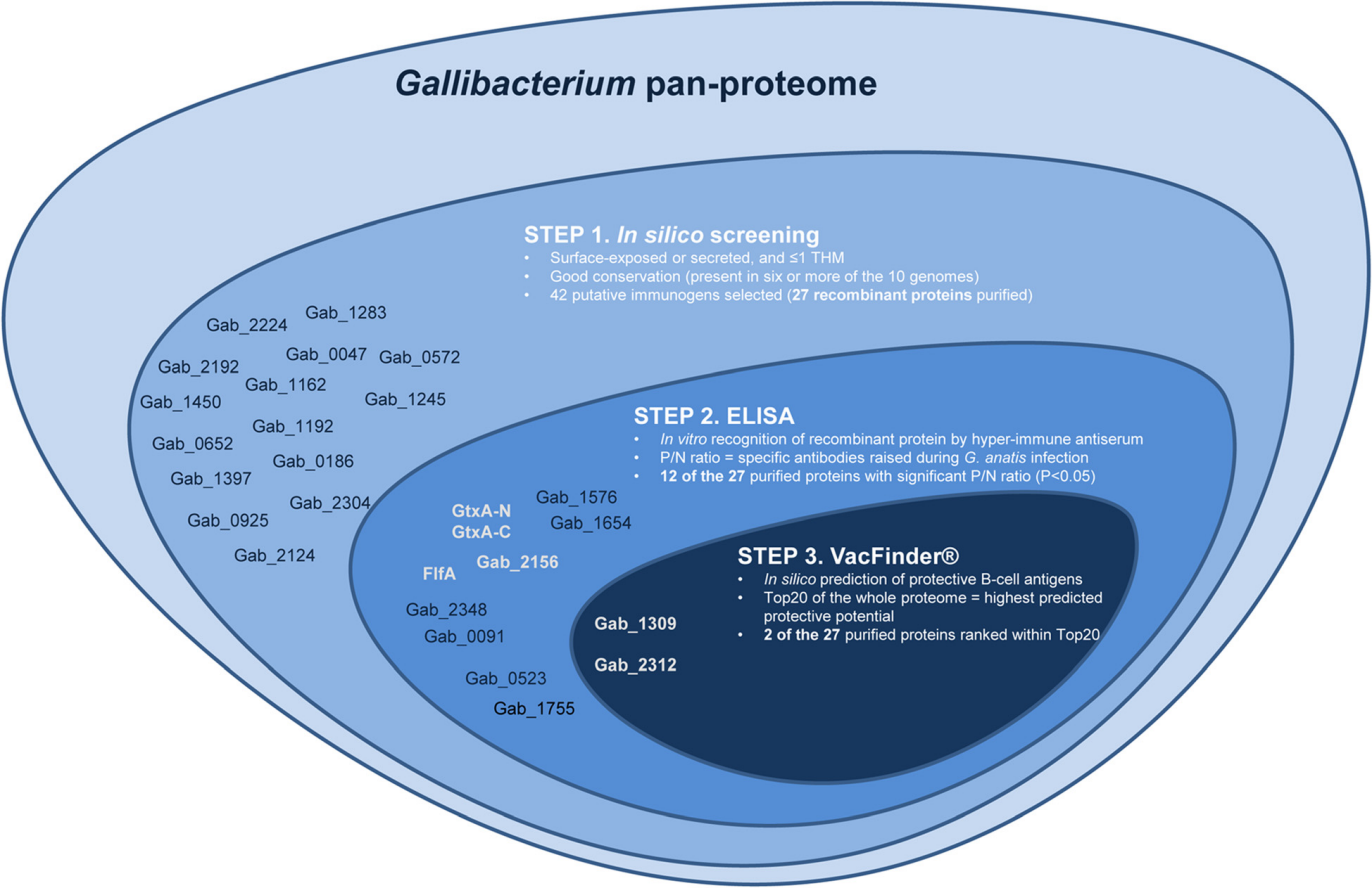
**Identification and selection of immunogens from** ***Gallibacterium.*** Summary of the three screening procedures used to identify and select putative immunogens from the *Gallibacterium* proteome. The 27 proteins successfully cloned, expressed and purified are listed and placed based on the screening results. Future investigations should focus on the proteins marked in white.

Novel prevention and treatment strategies are urgently needed to prevent *G. anatis* infections in the reproductive tract of chickens. In this study, a pan-genomic RV approach [15,23] was applied to identify novel and potentially broadly protective immunogens from *G. anatis*. The screening procedures and the main results are summarized in Figure 3. Of the 42 in silico predicted immunogens (Table 2), 27 proteins were successfully expressed in *E. coli* (Table 1), and of these, two novel proteins, Gab_1309 and Gab_2312, elicited a significant ELISA response and were also ranked in the Top20 of the *G. anatis* 12656*–*12 proteome by VacFinder®. Furthermore, these two proteins were present in all 10 strains included in this study as well as in the recently sequenced genome from *G. anatis* strain UMN179 [27].

Gab_1309 is a predicted lipoprotein, and this annotation is supported by the DOLOP database [50]. The protein sequence of Gab_1309 shows sequence similarity to the NlpD lipoprotein from *Yersinia pestis* [51]. By use of the Conserved Domain Database (CDD) feature in BLASTp [52], an N-terminal LysM domain and a C-terminal M23 peptidase domain can be identified in Gab_1309. The LysM domain is widespread among several bacterial species and is involved in peptidoglycan-binding and bacterial cell wall degradation [53,54], while the M23 family of endopeptidases is thought to be involved in bacterial cell wall separation [55]. Further studies are warranted to determine the specific function and role of Gab_1309 in *G. anatis* pathogenesis.

The C-terminal section of the second promising novel vaccine candidate identified in this study, Gab_2312, demonstrated 34% sequence similarity (conserved or identical residues) to the 200 kDa extracellular matrix protein adhesin A (EmaA) from *Aggregatibacter actinomycetemcomitans*. EmaA is widespread among *A. actinomycetemcomitans* strains [56] and belongs to the family of trimeric autotransporter adhesins (TAAs) [57]. TAAs are a group of homotrimeric virulence-related proteins in Gram-negative bacteria that primarily act as adhesins [58]. Interestingly, the Neisseria adhesin A (NadA) protein from MenB, which is one of the proteins in the recently approved 4CMenB vaccine (Bexsero®), also belongs to this family of TAAs [59], further suggesting a protective potential of Gab_2312. The prediction of Gab_2312 as a TAA protein was further confirmed using the recently developed domain annotation workflow of TAAs (daTAAs) [60].

Although Gab_1309 and Gab_2312 were the only proteins identified by both the ELISA screening and the in silico VacFinder® analysis, a number of proteins elicited a significant response as measured by ELISA, indicating that these proteins are expressed and immunogenic during *G. anatis* infection. It is currently unclear whether proteins that do not stimulate a natural immune response should be included as vaccine candidates. As our intraperitoneal immunization procedure bypassed the mucosal surface of e.g. the salpinx, this may not have induced a broad based natural immunological response, which in turn could explain why some proteins failed to induce a significant antibody response. However, studies have shown that high antigenicity of a protein does not necessarily lead to protection [61], questioning the validity of the antigen-specific titer as a marker of the overall ability to stimulate a protective immune response in vivo [62]. On the other hand, serological titers have been demonstrated to provide useful information about the reactivity of certain antigens to antibodies and thereby give indications about their potential as vaccine candidates [62]. In support of the ELISA antigen screening approach, three of the 10 proteins identified by the ELISA screening; GtxA, FlfA and Gab_2156, have recently been demonstrated as important, well-conserved *G. anatis* virulence-factors. GtxA is a large, cytolytic RTX toxin responsible for both the haemolytic and leukotoxic activity of *G. anatis* [26,35], while FlfA and Gab_2156 are both subunits of F17-like fimbriae [24]. To this, FlfA has been suggested to play a role in the tissue tropism of *G. anatis* by promoting adhesion to the epithelial lining in the reproductive tract and beyond during the typical course of disease [24]. The identification of GtxA, FlfA and Gab_2156 by ELISA further supports the predictive potential and importance of this screening approach, where particularly the N-terminal part of the GtxA-N elicited an extremely high antibody response in comparison to the remaining 26 proteins investigated (Figure 1 and Table 3), thereby suggesting that this part of the protein is particularly promising as a vaccine candidate. This suggestion is supported by a previous study by [63], demonstrating the protective potential of the N-terminal portion of the RTX protein, ApxI, against infections caused by *Actinobacillus pleuropneumoniae*.

The use of hyper-immune antiserum to screen for antigenic potential has previously been used successfully to identify putative immunogens from *Bacillus anthracis*, and in that study, the control sera (pre-immune antiserum) consisted of a mixture of naïve animal sera [64]. In our study, the pre-immune antiserum and hyper-immune serum was obtained from the same chickens before and after *G. anatis* 12656–12 infection. However, the chickens were found to be positive for *G. anatis* in the trachea and cloaca prior to infection. Since *G. anatis* is a typical part of the microflora in these organ systems [6], we find that this reflects the natural in vivo condition, which allows us to identify those proteins significantly expressed and exposed to the chicken immune system during infection. Thus, the ELISA screening applied in this study can be considered a semi-in vivo-evaluation and an important screening assay to evaluate antigenic potentials of recombinant proteins.

Of the 27 proteins tested by ELISA, 15 were not recognized by hyper-immune antiserum. However, these proteins might still possess immunogenic potential, as it is well-established that in vitro assessments may miss some proteins [14]. Moreover, a recent study focused interest on non-immunodominant protein regions for vaccine development and suggested the importance of analyzing major surface-exposed proteins for the presence of sub-regions that elicit protective immunity as a complement to the RV approach [65]. Additionally, the insolubility of many of the proteins tested by ELISA might also lead to false-negative titer-values, as it has been shown that linear epitopes are less likely to be immunodominant and elicit a good B-cell response if the protein possesses a higher-order structure [66]. On the other hand, the folding of a recombinant protein is not necessarily equivalent to the native structure of the protein, and moreover, folded recombinant proteins might expose epitopes normally hidden [14], and thereby lead to a false-positive ELISA response.

To provide an additional prediction of the protective potential, the sequences of 27 proteins were ranked within the *G. anatis* 12656–12 proteome by use of the in silico VacFinder® platform, in order to add an extra selective criterion. The ranking by VacFinder® is based on the predicted ability of a protein to elicit a high protective antibody response. Thus, the identified proteins are not affected by possible solubility problems or competing immunodominant epitopes. Moreover, VacFinder® does not consider general practicalities of cloning, expression and purification. The combination of VacFinder® prediction and ELISA screening identified two proteins, namely the above-mentioned Gab_1309 and Gab_2312. However, the previously identified and well-conserved virulence factors, GtxA, FlfA and Gab_2156, were not predicted as highly protective by VacFinder®. Leaving out GtxA, which is an exotoxin and not possible for VacFinder® to predict, FlfA and Gab_2156 were respectively ranked #58 and #41 out of the whole proteome. Hence, the Top20 of the proteome by VacFinder® is predicted to elicit the highest protective antibody response, yet this study demonstrates that proteins with a lower predicted protectiveness can be highly immunogenic.

Finally, the protective potential of the five most promising vaccine candidates; GtxA-N, FlfA, Gab_1309, Gab_2156 and Gab_2312, was evaluated in vivo. The results from these preliminary in vivo immunization trials indicated that at least GtxA-N, FlfA and Gab_1309 are promising vaccine candidates with a good protective potential. These results correspond well with previous results demonstrating the in vivo protective potential of FlfA [24]. Further studies, including larger group sizes, different doses and repeated immunization, are needed to confirm the protective potential of the recombinant proteins. Moreover, the ability of the recombinant proteins to elicit a broadly cross-protective immune response against heterologous strains should also be investigated. Still, the results presented in this paper provide an important step towards the development of a new and broadly protective *G. anatis* vaccine.

## Additional files

Additional file 1:
***E. coli*** **strains and plasmids.** *E. coli* strains and plasmid used in this study to clone and express recombinant proteins [67]. Additional file 2:
**Cloning and protein expression.** Detailed description of the methods used for the cloning as well as the small scale and large-scale protein expression and purification [37,38,68-70]. Additional file 3:
**Primer sequences, PCR products and expression vectors.** A table summarizing the primer sequences used for gene amplification, the size of the resulting PCR product and the final expression vector chosen for protein expression from each of the *G. anatis* 12656*–*12 genes that were successfully small-scaled cloned and expressed [24,35,36]. Additional file 4:
**Multiple sequence alignment of Gab_1309.** A multiple alignment between Gab_1309 and homologs was conducted using MAFFT (v7.130b) [45] and formatted using Jalview 2.8.0b1 [46]. Amino acids were colored in blue based on their conservation (dark blue = fully conserved). Additional file 5:
**Multiple sequence alignment of recombinant Gab_2312.** A multiple alignment between the 338-residue recombinant Gab_2312 protein sequence and Gab_2312 protein homologs was conducted using MAFFT (v7.130b) [45] and formatted using Jalview 2.8.0b1 [46]. Amino acids were colored in blue based on their conservation (dark blue = fully conserved). The two head domains included in the recombinant protein, which were predicted by the domain annotation workflow of TAAs (daTAAs) [60], are marked with red and green boxes, respectively.

## Abbreviations

RV: Reverse vaccinology
TMH: Trans-membrane helix
*G. anatis* 12656–12: *Gallibacterium anatis* biovar *haemolytica* strain 12656–12
MenB: *Neisseria meningitides* seropgroup B
LIC: Ligation-independent cloning
ELISA: Enzyme-linked immunosorbent assay
TAA: Trimeric autotransporter adhesin
