# Supplementary material for: In silico prediction of Gallibacterium anatis pan-immunogens
Source: Vet Res. 2014 Aug 8;45(1):80. doi: 10.1186/s13567-014-0080-0 (PMC4423631; doi:10.1186/s13567-014-0080-0)
Supplement: Additional file 1: — E. coli strains and plasmids. E. coli strains and plasmid used in this study to clone and express recombinant proteins [67]. [file 13567_2014_80_MOESM1_ESM.docx]

| **Strains/Plasmids** | | **Relevant characteristics*^a^*** | **Reference or source** |
| --- | --- | --- | --- |
| *E. coli* strains | |  |  |
|  | TOP10 | *E. coli* strain used for Gateway cloning | Invitrogen |
|  | DH5α | *E. coli* strain used for Gateway cloning | Invitrogen |
|  | BL21 CodonPlus | *E. coli* strain used for small scale expression of recombinant proteins in pDEST vectors | Stratagene |
|  | Mach1 | *E. coli* strain used for LIC cloning | Invitrogen |
|  | Rosetta 2 (DE3) | BL21-derived *E. coli* strain used for small scale expression of recombinant proteins in the pNIC28-Bsa4 vector, as well as scale-up expression of all recombinant proteins | Novagen |
| Plasmids | |  |  |
|  | pENTR^TM^/SD/D-TOPO® | Directional cloning vector for entry to the Gateway® System | Invitrogen |
|  | pDEST17 | Gateway® Destination vector (Amp^R^, N-terminal His_6_ fusion tag, TEV cleavable) | Invitrogen |
|  | pDEST41BA | Gateway® Destination vector (Amp^R^, N-terminal His_6_ and NusA fusion tag, TEV cleavable) | [67] |
|  | pNIC28-Bsa4 | pET28a-derived vector for LIC cloning and expression (Kan^R^, N-terminal His_6_ fusion tag, TEV cleavable) | GenBank accession EF198106 |

*^a^*Amp, ampicillin; His, histidin; Km, kanamycin. Superscript “R” designates resistance.

^T^= type strain
