# Supplementary material for: In silico prediction of Gallibacterium anatis pan-immunogens
Source: Vet Res. 2014 Aug 8;45(1):80. doi: 10.1186/s13567-014-0080-0 (PMC4423631; doi:10.1186/s13567-014-0080-0)
Supplement: Additional file 2: — Cloning and protein expression. Detailed description of the methods used for the cloning as well as the small scale and large-scale protein expression and purification [37,38,68-70]. [file 13567_2014_80_MOESM2_ESM.docx]

**Additional file 2 Cloning and protein expression**

Each of the selected genes was amplified from the *G. anatis* 12656-12 genome by PCR and cloned into the Gateway entry vector pENTR^TM^/SD/D-TOPO (Invitrogen). Primers were designed using Oligo Explorer 1.2 (Gene Link^TM^, Hawthorne, NY, USA) as described previously [37]. Areas with high predicted hydrophobicity in the N and/or C terminus were removed, as were predicted signal peptides. In addition, GtxA was cloned as two parts (N- and C-terminal) due to its size. Each gene was then transferred from the entry vector into the Gateway destination vectors pDEST17 (His_6_ fusion tag) and pDEST41BA (His_6_ and NusA fusion tag) and used to transform each of the *E. coli* expression strains as listed in Table 3. Small-scale protein expression from each of the recombinant expression constructs was carried out using the Overnight Express System (Novagen, Madison, WI, USA). Briefly, single colonies were inoculated in Overnight Express System medium containing 100 µg/mL ampicillin (Sigma-Aldrich, St Louis, MO, USA) and incubated for approximately 16 h at 37 °C, 200 rpm. Cells were harvested by centrifugation, resuspended in PBS and lysed by sonication. The supernatant (soluble proteins) and pellet (insoluble proteins) fractions from each expression were analyzed by SDS-PAGE to assess expression levels and solubility of each fusion protein.

From the 42 proteins initially selected, 37 expression constructs were generated. However, 15 of these constructs resulted in low protein yields, unstable protein products and/or insoluble NusA-tagged fusion proteins. Consequently, these 15 genes were re-cloned into the expression vector pNIC28-Bsa4 (His_6_ fusion tag) using the ligation-independent cloning (LIC) strategy [68]. The recombinant proteins were expressed on a small-scale and purified as described previously [69] with the following modifications. Starter cultures were prepared in Terrific Broth (TB) medium supplemented with 50 µg/mL kanamycin and 25 µg/mL chloramphenicol (Sigma-Aldrich). Expression cultures, prepared from the starter cultures, were incubated for 1.5 h at 18 °C before adding 0.1 mM isopropyl-β-D-1-thiogalactopyranoside (IPTG) to induce protein expression. Cell pellets were resuspended in the supplemented lysis buffer by shaking at 1000 rpm for 10 min, followed by incubation with continual mixing at 500 rpm for 40 min. An aliquot of cell lysate (containing all proteins) was stored for subsequent analysis by SDS-PAGE, while remaining cell lysate was centrifuged for 30 min at 2470 × *g* at 4 °C and supernatants (containing soluble proteins only) were removed to a new deep well plate for subsequent purification.

All proteins were expressed and purified in large-scale from *E. coli* Rosetta 2 (DE3) cells (Novagen). Large-scale expression was performed in a custom-made large-scale expression system (LEX) (Harbinger Biotech, Toronto, Canada) as described previously [38] with the following modifications. Starter cultures were prepared in TB medium supplemented with 50 µg/mL kanamycin and 25 µg/mL chloramphenicol (Sigma-Aldrich) and incubated overnight at 37 °C with 180 rpm shaking. Each overnight culture was diluted 100 times in 1.5 L pre-heated TB medium supplemented with 50 µg/mL kanamycin and 200 µL polypropylenglycol 2000. Following protein expression, cells were harvested by centrifugation and resuspended in lysis buffer (50 mM NaP, 300 mM NaCl, 0.5 mM Tris (2-carboxyethyl) phosphine (TCEP), 10% glycerol; pH 7.5) supplemented with Complete Mini EDTA-free protease inhibitor (Roche) and 50 U/mL Benzonase (Sigma-Aldrich). Cells were lysed by three passages through a high-pressure homogenizer (EmulsiFlex-D20, Avestin, Mannheim, Germany) at 1000 Bar.

For soluble proteins, cell lysates were centrifuged at 18 500 × *g* for 30 min at 4 °C and supernatants filtered through a 0.22 µm PES bottle top filter (TPP MidSci, Valley Park, MO, USA). The proteins were purified by affinity chromatography on an ÄKTA Xpress System on HiTrap Chelating columns (GE Healthcare, Buckinghamshire, UK) charged with Ni^2+^. Columns were equilibrated with binding buffer (50 mM NaP, 300 mM NaCl, 0.5 mM TCEP, 10% glycerol, 10 mM imidazole; pH 7.5) prior to loading of samples. Columns were washed with binding buffer until a stable A_280_ baseline was reached, and a subsequent stringency wash with wash buffer (50 mM NaP, 300 mM NaCl, 0.5 mM TCEP, 10% glycerol, 30 mM imidazole; pH 7.5) was applied to wash out low affinity binding proteins. Proteins were step-eluted with 100% elution buffer (50 mM NaP, 300 mM NaCl, 0.5 mM TCEP, 10% glycerol, 500 mM imidazole; pH 7.5) and further purified by use of size exclusion chromatography (SEC) on HiLoad 16/60 Superdex 75 pg or 16/60 Superdex 200 pg gel filtration columns equilibrated with SEC buffer (50 mM NaP, 150 mM NaCl, 0.5 mM TCEP, 10% glycerol; pH 7.5). Peak fractions of 2 mL were collected automatically.

For insoluble proteins, cell lysates were centrifuged at 18 500 × *g* for 30 min at 4 °C and the pellets resuspended in Triton wash buffer (100 mM NaP, 150 mM NaCl, 0.5 mM TCEP, 1% v/v Triton X-100 100% (Sigma-Aldrich); pH 7.5). Samples were then centrifuged (18 500 *g*, 30 min, 20 °C), resuspended in inclusion body resuspension buffer (100 mM NaP, 150 mM NaCl, 0.5 mM TCEP, 6M guanidinium HCl (Sigma-Aldrich); pH 7.5), centrifuged (18 500 *g*, 1 h, 20 °C), and filtered through a 0.22 µm PES bottle top filter (TPP MidSci, MO). The insoluble proteins were purified by affinity chromatography as described for soluble proteins except that running buffer (100 mM NaP, 150 mM NaCl, 0.5 mM TCEP, 6M Urea (Merck, Whitehouse Station, NJ); pH 7.5) was used instead of binding and wash buffer, and insoluble elution buffer (100 mM NaP, 150 mM NaCl, 0.5 mM TCEP, 0.5M imidazole, 6M Urea (Merck); pH 7.5) was used to elute the fusion proteins. Peak fractions of 1.5 mL were collected automatically.

All the collected peak fractions were analyzed by SDS-PAGE. Relevant fractions were pooled and protein concentration was assessed with a Nanodrop ND-1000 spectrophotometer (NanoDrop Technologies, Wilmington, DE, USA) using the theoretical mass extinction coefficient for the relevant protein as calculated with the ProtParam tool [70]. Protein purity was determined using ImageQuant (GE Amersham, UK). Soluble proteins were stored at −80 °C, while insoluble proteins were stored at 4 °C.
