# Supplementary material for: In silico prediction of Gallibacterium anatis pan-immunogens
Source: Vet Res. 2014 Aug 8;45(1):80. doi: 10.1186/s13567-014-0080-0 (PMC4423631; doi:10.1186/s13567-014-0080-0)
Supplement: Additional file 3: — Primer sequences, PCR products and expression vectors. A table summarizing the primer sequences used for gene amplification, the size of the resulting PCR product and the final expression vector chosen for protein expression from each of the G. anatis 12656–12 genes that were successfully small-scaled cloned and expressed [24,35,36]. [file 13567_2014_80_MOESM3_ESM.docx]

| **Protein ID** | **Primers** | **Primer sequences^a^** | **Size (bp)** | **Expression vector** |
| --- | --- | --- | --- | --- |
| Gab_0001 | Fw | 5´-**caccatg**caactcactgccgaccaagattc-3’ | 1341 | pDEST41BA |
|  | Rv | 5´-**tca**attcaaaccagcctgataattaag-3’ |  |  |
| Gab_0047 | Fw | 5´- TACTTCCAATCCATGAAAGTTTTAGGAATAGAAAGCTCCTGCGATGAAAC-3’ | 1047 | pNIC28-Bsa4 |
|  | Rv | 5´-TATCCACCTTTACTGTTAGCTAACAATCTTCGCTAAATCACTCATTTTCC-3’ |  |  |
| Gab_0087 | Fw | 5´-TACTTCCAATCCATGCCTTTACAAGAAAATATCTTGCAAGTAAAACAAGAAAACG-3’ | 1479 | pNIC28-Bsa4 |
|  | Rv | 5´-TATCCACCTTTACTGTTAGCTAGAATTATCGTAAGATTTAAAGGCTCTTGC-3’ |  |  |
| Gab_0091 | Fw | 5´-TACTTCCAATCCATGCTCTACAGCGGCAGTGTATATAGCG-3’ | 432 | pNIC28-Bsa4 |
|  | Rv | 5´-TATCCACCTTTACTGTTAAAGTACGGAAACGTTTAGAGAACCGCC-3’ |  |  |
| Gab_0178 | Fw | 5´-**caccatg**agcacccaacaaaatacggaac-3’ | 759 | pDEST17 |
|  | Rv | 5´-ttatttaccatcccacgcttttaatg-3’ |  |  |
| Gab_0186 | Fw | 5´-**caccatg**cagcatatctcttccagagg-3’ | 1011 | pDEST17 |
|  | Rv | 5´-**tca**ccaaactcgtccataatgttttaataatc-3’ |  |  |
| Gab_0337 | Fw | 5´-**caccatg**gcagagccaaaagcgattac-3’ | 3483 | pDEST17 |
|  | Fw | 5´-**tca**agtaaagtcgttttgcgtattatg-3’ |  |  |
| Gab_0523 | Fw | 5´-**caccatg**tctgataaagcagctgatcaac-3’ | 378 | pDEST41BA |
|  | Rv | 5´-**tca**taatactgcacgacgatctttag-3’ |  |  |
| Gab_0572^b^ | Fw | 5´-TACTTCCAATCCATGGACGGAACAGTTAATTTTAATGGGAAATTGGTAG-3’ | 498 | pNIC28-Bsa4 |
|  | Rv | 5´-TATCCACCTTTACTGTTATTCGTATTCGACATAGTAATCTACTGCGGC-3’ |  |  |
| Gab_0574 | Fw | 5´-TACTTCCAATCCATGAGCGTAGAAGCCGTTGAATTTGCTGAG-3’ | 2412 | pNIC28-Bsa4 |
|  | Rv | 5´-TATCCACCTTTACTGTTAGGAAGTTTTATGATTAATGTGATAATGAATTACGCATTG-3’ |  |  |
| Gab_0602 | Fw | 5´-**caccatg**gcagcgaatagccaaaatac-3’ | 1191 | pDEST17 |
|  | Rv | 5´-ctatttaagatcagtagcccatttg-3’ |  |  |
| Gab_0652 | Fw | 5´-**caccatg**accggtgaggtggtgaaaac-3’ | 2238 | pDEST17 |
|  | Rv | 5´-Ctatctcaaactaaacggatcg-3’ |  |  |
| Gab_0925 | Fw | 5´-**caccatg**tttgatagcgaatcgaaacc-3’ | 2343 | pDEST17 |
|  | Rv | 5´-**tca**tgtgtatcctccatcaaatttataaac-3’ |  |  |
| Gab_0999 | Fw | 5´-**caccatg**cactttgctaatgaccaactttc-3’ | 426 | pDEST41BA |
|  | Rv | 5´-Ttagtattgttgttggatactgatc-3’ |  |  |
| Gab_1008 | Fw | 5´-**caccatg**agttatgcttacgcgaatgag-3’ | 2436 | pDEST17 |
|  | Rv | 5´-ttaataaaattggcattgtactggc-3’ |  |  |
| Gab_1162 | Fw | 5´-**caccatg**tgtgcaagccatcaggataatg-3’ | 672 | pDEST17 |
|  | Rv | 5´-**tca**atctaaaatatttttatccagcttctc-3’ |  |  |
| Gab_1245 | Fw | 5´-TACTTCCAATCCATGGGTGATGATATTCAAGCATTTCCCTCGG-3’ | 1617 | pNIC28-Bsa4 |
|  | Rv | 5´-TATCCACCTTTACTGTTAGAATTGATAGCTTAAATTAAATCCAGTTGTAACGTGC-3’ |  |  |
| Gab_1283 | Fw | 5´-TACTTCCAATCCATGACTTCCGGTTTAGGCCGTGCTTATG-3’ | 1221 | pNIC28-Bsa4 |
|  | Rv | 5´-TATCCACCTTTACTGTTATAATGAACCTTGCTTCTCATCAAAAGATAAGTTTTTACC-3’ |  |  |
| Gab_1309 | Fw | 5´-**caccatg**agtaataattctgcgcctgtc-3’ | 1155 | pDEST17 |
|  | Rv | 5´-**tca**atttcttggtaggtaattggttg-3’ |  |  |
| Gab_1397 | Fw | 5´-**caccatg**gataaaccgataaccttaaaag-3’ | 1644 | pDEST41BA |
|  | Rv | 5´-**tca**ttggatatttttactgtcgtctttatc-3’ |  |  |
| Gab_1450 | Fw | 5´-TACTTCCAATCCATGGATGGGGAGCTTAATAAAAGTAAAGTGACTCC-3’ | 477 | pNIC28-Bsa4 |
|  | Rv | 5´-TATCCACCTTTACTGTTATTTGCCAAGAATATTATATTCCGCCGCAACATC-3’ |  |  |
| Gab_1576 | Fw | 5´-**caccatg**gtggcaaaaaataaagaaatttc-3’ | 594 | pDEST17 |
|  | Rv | 5´-**tca**accaaccttcacattgcgac-3’ |  |  |
| Gab_1631 | Fw | 5´-TACTTCCAATCCATGACACCTAAAAGACCAGCAGTTGAAACTGC-3’ | 1056 | pNIC28-Bsa4 |
|  | Rv | 5´-TATCCACCTTTACTGTTATTTTTTATAGATATAGGTACCGTTGCTTTGGCG-3’ |  |  |
| Gab_1654 | Fw | 5´-**caccatg**agtttattaccttaccaaag-3’ | 753 | pDEST17 |
|  | Rv | 5´-**tca**taaaccgatgcgatcggtgg-3’ |  |  |
| Gab_1755 | Fw | 5´-**caccatg**tgtggaaatttaagtaaagttag-3’ | 678 | pDEST17 |
|  | Rv | 5´-**tca**gatcacaacacgacgattcg-3’ |  |  |
| Gab_2156^b^ | Fw | 5´-TACTTCCAATCCATGAATAATCCACCAGCCCAACACGGC-3’ | 528 | pNIC28-Bsa4 |
|  | Rv | 5´-TATCCACCTTTACTGTTAACCAGTAGTAATTTGTGCAGGCTCCTTG-3’ |  |  |
| Gab_2158 | Fw | 5´-TACTTCCAATCCATGAGTGACTACGTTGAATTTGATTCTGATTTTTTATACGG-3’ | 2451 | pNIC28-Bsa4 |
|  | Rv | 5´-TATCCACCTTTACTGTTAATAAAACTGGCATTGTATTGGCACGATAAGTAATTTAC-3’ |  |  |
| Gab_2192 | Fw | 5´-**caccatg**aataccgatactcacaactataaaag-3’ | 546 | pDEST17 |
|  | Rv | 5´-**tca**agtaacacgtgcttcttgagag-3’ |  |  |
| Gab_2224 | Fw | 5´-**caccatg**gaaatgttagaggaaatagaag-3’ | 2076 | pDEST17 |
|  | Rv | 5´-**tca**gctcactttcacatttcgcc-3’ |  |  |
| Gab_2304 | Fw | 5´-**caccatg**ggcacacaaagcgtatcctg-3’ | 1035 | pDEST17 |
|  | Rv | 5´-ctatttcttgcactgatttgctaag-3’ |  |  |
| Gab_2312 | Fw | 5´-**caccatg**ttggcagatcaaccacaagc-3’ | 1014 | pDEST17 |
|  | Rv | 5´-**tca**ttctgaaccgtttttactggttttc-3’ |  |  |
| Gab_2347 | Fw | 5´-TACTTCCAATCCATGAGCACGACAACGTTTGCTGCACCG-3’ | 2265 | pNIC28-Bsa4 |
|  | Rv | 5´-TATCCACCTTTACTGTTAATCCGGTACGCCTTTAAGCGGGAATG-3’ |  |  |
| Gab_2348 | Fw | 5´-**caccatg**atgaaaaaatcattaaaagttg-3’ | 534 | pDEST17 |
|  | Rv | 5´-ctattttttatctgctgccgg-3’ |  |  |
| FlfA^b^ | Fw | 5´-TACTTCCAATCCATGGATGATCCTAGTGCAGCTAATTCAACAAATGG-3’ | 507 | pNIC28-Bsa4 |
|  | Rv | 5´-TATCCACCTTTACTGTTACGGATTAGTTGCATAGTAACGAGCTTTATATTTC-3’ |  |  |
| OmpC^c^ | Fw | 5´-**caccatg**gtagtttataatcaagacgg-3’ | 1053 | pDEST17 |
|  | Rv | 5´-**tca**aacgccgattttgttatctctg-3’ |  |  |
| GtxA-N^d^ | Fw | 5´-TACTTCCAATCCATGCTTTCATTAAAAGAAAAAGTAACTGGAATAGATTTTGATGC-3’ | 3367 | pNIC28-Bsa4 |
|  | RV | 5´-TATCCACCTTTACTGTTAAGGATTGCCGTCTTTGCCTACTGTTTTC |  |  |
| GtxA-C^d^ | Fw | 5´-TACTTCCAATCCATGGGCTTTATCACTGATGTGGTGAAAACAGTAG | 2869 | pNIC28-Bsa4 |
|  | RV | 5´-TATCCACCTTTACTGTTAATTGCCGGAAGTAATATTAACATCGCCTCC |  |  |

^a^ Primers for cloning by use of the Gateway system (the resulting overhangs are marked in bold) or the LIC strategy (the resulting overhangs are underlined).

^b^ Previously described in [24].

^b^ Previously described in [36].

^c^ Previously described in [35]. Due to the size of the protein it was cloned, expressed and purified as two halves.

Fw = forward primer.

Rv = reverse primer.
