# Supplementary material for: In silico prediction of Gallibacterium anatis pan-immunogens
Source: Vet Res. 2014 Aug 8;45(1):80. doi: 10.1186/s13567-014-0080-0 (PMC4423631; doi:10.1186/s13567-014-0080-0)
Supplement: Additional file 4: — Multiple sequence alignment of Gab_1309. A multiple alignment between Gab_1309 and homologs was conducted using MAFFT (v7.130b) [45] and formatted using Jalview 2.8.0b1 [46]. Amino acids were colored in blue based on their conservation (dark blue = fully conserved). [file 13567_2014_80_MOESM4_ESM.pdf]

```
   Avicor MKQSFLLIPVVAAVLAGCSSNNSAPVEDVDGTLTPGIMQSVGNSANSTWEPQVQQQQMPSDMMATPAA
     4895 MKQSFLLIPVVAAVLAGCSSNNSAPVEDVDGTLTPGIMQSVGNSANSTWEPQVQQQQMPSDMMATPTA
  10672-6 MKQSFLLIPVVAAVLAGCSSNNSAPVEDVDGTLTPGIMQSVGNSANSTWEPQVQQQQMPSDMMATPTA
     F149 MKQSFLLIPVVAAVLAGCSSNNSAPVEDVDGTLTPGIMQSVGNSANSTWEPQVQQQQMPSDMMATPAA
     7990 MKQSFLLIPVVAAVLVGCSSNNSAPVEDVDGTLTPGIMQSVGNSANSTWEPQVQQQQMPSDMMATPAA
     IPDH MKQSFLLIPVVAAVLAGCSSNNSAPVEDVNGTLTPGIMQSVGNSANSTWEPQVQQQQMPSDMMATPAA
 12656-12 MKQSFLLIPVVAAVLTGCSSNNSAPVEDVDGTLTPGIMQSVGNSANSTWEPQVQQQQMPSDMMATPAA
  CCM5995 MKQSFLLIPVVAAVLAGCSSNNSAPVEDVDGTLTPGIMQSVGNSANSNWEPQVQQQQMPSDMMAAPQ-
  CCM5976 MKQSFLLIPVVAAVLAGCSSNNSAPVEDVDGTLTPGIMQSVGNSANSNWEPQVQQQQMPSDMMAAPQ-
  CCM5974 MKQSFLLIPVVVAVLAGCSSNNSAPVEDVNGTLTPGIMQSVGNSANSTWEPQVQQQQMPSDMMATPAT

   Avicor PQPQAQPVQSQPVQTQPTYQQPQPVVAPTPAPQPVTKPAAKKAVSQDFTIPRNPQTNAPDYSKIDKGF
     4895 PQPQAQPVQSQPVQTQPTYQQPQPVAAPTPAPQPVTKPAAKKAVSQDFTIPRNPQTNAPDYSKIDKGF
  10672-6 PQPQAQPVQSQPVQTQPTYQQPQPVAAPTPAPQPVTKPAAKKAVSQDFTIPRNPQTNAPDYSKIDKGF
     F149 PQPQAQPVQSQPVQTQPTYQQPQPVAAPTPAPQPVTKPAAKKAVSQDFTIPRNPQTNAPDYSKIDKGF
     7990 PQPQAQPVQSQPVQTQPTYQQPQPVAAPTPAPQPVTKPAAKKAVSQDFTIPRNPQTNAPDYSKIDKGF
     IPDH PQPQAQPVQSQPVQTQPTYQQPQPVSAPTPAPQPVTKPATKKAVSQDFTIPRNPQTNAPDYSKIDKGF
 12656-12 PQPQAQPVQSQPVQTQPTYQQPQPIAAPTPAPQPVTKPATKKAISQDFTIPRNPQTNAPDYSKIDKGF
  CCM5995 -------TQTQPVQTQPTYQQPQPVSAPTPAPQPVTKPAAKKAVSQDFTIPRNPQTNAPDYSKIDKGF
  CCM5976 -------TQTQPVQTQPTYQQPQPVSAPTPAPQPVTKPATKKAVSQDFTIPRNPQTNAPDYSKIDKGF
  CCM5974 PQP-----QAQPVQTQSTYQQPQPVSAPTPAPQPVTKPATKKAVSQDFTIPRNPQTNAPDYSKIDKGF

   Avicor YKGDSYTVRKGDTMFLIAYISGMDVRELAALNHIPEPYKLSVGQKLRISNNAAESNETIAATTTTAST
     4895 YKGDSYTVRKGDTMFLIAYISGMDVRELAALNHIPEPYKLSVGQKLRISNNATESNETIAATTTTTST
  10672-6 YKGDSYTVRKGDTMFLIAYISGMDVRELAALNHIPEPYKLSVGQKLRISNNATESNETVAATTTTAST
     F149 YKGDSYTVRKGDTMFLIAYISGMDVRELAALNHIPEPYKLSVGQKLRISNNAAESNETIAATTTTTST
     7990 YKGDSYTVRKGDTMFLIAYISGMDVRELAALNHIPEPYKLSVGQKLRISNNAAESNETIAATTTTAST
     IPDH YKGDSYTVRKGDTMFLIAYISGMDVRELAALNHIPEPYKLSVGQKLRISNNAAESNETAAATTTTAST
 12656-12 YKGDSYTVRKGDTMFLIAYISGMDVRELAALNHIPEPYKLSVGQKLRISNNVAESNETVAATTTTAST
  CCM5995 YKGDSYTVRKGDTMFLIAYISGMDVRELAALNHIPEPYKLSVGQKLRISNNATESNETIAATTTTTST
  CCM5976 YKGDSYTVRKGDTMFLIAYISGMDVRELAALNHIPEPYKLSVGQKLRISNNAAESNETITATTTTAST
  CCM5974 YKGDSYTVRKGDTMFLIAYISGMDVRELAALNHIPEPYKLSVGQKLRISNNAAESNETIAATTTTAST

   Avicor TTAAAAPAQPAVTYTPGPNGTAYGSDGTVIGPIKSTAGTMPTTSNNSVNTVPVEPQPTTTVNQAPRNV
     4895 TTAAAAPAQPAVTYTPGPNGTAYGSDGTVIGPIKSTAGTMPTTSNNSVNTVPVEPQPTTTVNQAPRNV
  10672-6 TTAAAAPAQPAVTYTPGPNGTAYGSDGTVIGPIKSTAGTMPTTSNNSVNTVPVEPQPTTTVNQAPRNV
     F149 TTAAAAPAQPAVTYTPGPNGTAYGSDGTVIGPIKSTAGTMPTTSNNSVNTVPVEPKPTTTVNQAPRNV
     7990 TTAATAPAQPAVTYTPGPNGTAYGSDGTVIGPIKSTAGTMPTTSNNSVNTVPVEPQPTTTVNQAPRNV
     IPDH TTASAAPAQPAVTYTPGPNGTAYGSDGTVIGPIKSTAGTMPTTSNNSVNTVPVEPQPTTTVNQAPRNV
 12656-12 TTAAAAPAQPAVTYTPGPNGTAYGSDGKVIGPIKSTAGTMPTTSNNSVNTVPVEPQPTTTVNQAPRNV
  CCM5995 TTAAAAPAQPAVTYTPGPNGTAYGSDGTVIGPIKSTAGTMPTTSNNSVNTVPVEPQPTTTVNQAPRNV
  CCM5976 TTAATAPTQPAVTYTPGPNGTAYGSDGTVIGPIKSTAGTMQTTSNNSVNTVPVEPQPTNTVNQAPRNI
  CCM5974 TTAATAPTQPAVTYTPGPNGTAYGSDGTVIGPIKSTAGTMQTTSNNSVNTVPVEPQPTNTVNQAPRNI

   Avicor EPVAVNNSNITWIWPAKGNIIQGFSNSDGGNKGIDIGGSRGQAVYAAAPGRVVYAGNALRGYGNLIII
     4895 EPVAVNNSNITWIWPAKGNIIQGFSNSDGGNKGIDIGGSRGQAVYAAAPGRVVYAGNALRGYGNLIII
  10672-6 EPVAVNNSNITWIWPAKGNIIQGFSNSDGGNKGIDIGGSRGQAVYAAAPGRVVYAGNALRGYGNLIII
     F149 EPVAVNNSNITWIWPAKGNIIQGFSNSDGGNKGIDIGGSRGQAVYAAAPGRVVYAGNALRGYGNLIII
     7990 EPVAVNNSNITWIWPAKGNIIQGFSNSDGGNKGIDIGGSRGQAVYAAAPGRVVYAGNALRGYGNLIII
     IPDH EPVAVNNSNITWIWPVKGNIIQGFSNSDGGNKGIDIGGSRGQAVYAAAPGRVVYAGNALRGYGNLIII
 12656-12 EPVAVNNSNITWIWPAKGNIIQGFSNSDGGNKGIDIGGSRGQAVYAAAPGRVVYAGNALRGYGNLIII
  CCM5995 EPVAVNNSNITWIWPAKGNIIQGFSNSDGGNKGIDIGGSRGQAVYAAAPGRVVYAGNALRGYGNLIII
  CCM5976 EPVAVNNSNITWIWPTKGNIIQGFSNSDGGNKGIDIGGSRGQAVYAAAPGRVVYAGNALRGYGNLIII
  CCM5974 EPVAVNNSNITWIWPTKGNIIQGFSNSDGGNKGIDIGGSRGQAVYAAAPGRVVYAGNALRGYGNLIII

   Avicor KHNDDYLSAYAHNESILVKDQQQVTAGQQIAKMGSSGTNSVKLHFEIRYKGKSVNPTNYLPRN
     4895 KHNDDYLSAYAHNESILVKDQQQVTAGQQIAKMGSSGTNSVKLHFEIRYKGKSVNPTNYLPRN
  10672-6 KHNDDYLSAYAHNESILVKDQQQVTAGQQIAKMGSSGTNSVKLHFEIRYKGKSVNPTNYLPRN
     F149 KHNDDYLSAYAHNESILVKDQQQVTAGQQIAKMGSSGTNSVKLHFEIRYKGKSVNPTNYLPRN
     7990 KHNDDYLSAYAHNESILVKDQQQVTAGQQIAKMGSSGTNSVKLHFEIRYKGKSVNPTNYLPRN
     IPDH KHNDDYLSAYAHNESILVKDQQQVTAGQQIAKMGSSGTNSVKLHFEIRYKGKSVNPTNYLPRN
 12656-12 KHNDDYLSAYAHNESILVKDQQQVTAGQQIAKMGSSGTNSVKLHFEIRYKGKSVNPTNYLPRN
  CCM5995 KHNDDYLSAYAHNESILVKDQQQVTAGQQIAKMGSSGTNSVKLHFEIRYKGKSVNPTNYLPRN
  CCM5976 KHNDDYLSAYAHNESILVKDQQQVTAGQQIAKMGSSGTNSVKLHFEIRYKGKSVNPTNYLPRN
  CCM5974 KHNDDYLSAYAHNESILVKDQQQVTAGQQIAKMGSSGTNSVKLHFEIRYKGKSVNPTNYLPRN
```
