# Supplementary material for: In silico prediction of Gallibacterium anatis pan-immunogens
Source: Vet Res. 2014 Aug 8;45(1):80. doi: 10.1186/s13567-014-0080-0 (PMC4423631; doi:10.1186/s13567-014-0080-0)
Supplement: Additional file 5: — Multiple sequence alignment of recombinant Gab_2312. A multiple alignment between the 338-residue recombinant Gab_2312 protein sequence and Gab_2312 protein homologs was conducted using MAFFT (v7.130b) [45] and formatted using Jalview 2.8.0b1 [46]. Amino acids were colored in blue based on their conservation (dark blue = fully conserved). The two head domains included in the recombinant protein, which were predicted by the domain annotation workflow of TAAs (daTAAs) [60], are marked with red and green boxes, respectively. [file 13567_2014_80_MOESM5_ESM.pdf]

```
7990                ----QGAKPQNVTPT-----DVKNNFVNPN------GTDSIVLDNVKSGIGGE----VKD--
CCM5995             ----PDAKPQTIETA-----NIKNKMVNPN------SNDSIVLDNVKSGIGGK----VVDPE
IPDH                ----PGAQPQNVTPT-----DVKNNFVNPN------GTGSIVLDNVKSGIGGK----VVDPE
10672-6             ----KDKSPLAEQPIALDQSKLKNSVVNPN------GDGATIVDNVKSGIGGE----VKD--
CCM5974             ------KDAQAVTPNPVDQSNVKTSLVNPNTGVGTGTDTSMTLDNVKSGIGGKAGNGIKDGT
F149                --------------------------------------------------------------
4895                --------PDA-KPQTIETANIKNKMVNPN------SNDSIVLDNVKSGIGGK----VVDPE
Avicor              DDGITPKQPLADQPQAVDQNTLKNSVVNPN------GNGSTVLDNVKSGIGGDAGNGIKNGS
CCM5976             DDGITPKQPLADQPQAVDQNTLKNSVVNPN------GNGPTVLDNVKSGIGGDAGNGIKNGS
RecGab_2312(aa1-91) ---------LADQPQAVDQNMLKNSVVNPN------GNDPTVLDNVKSGIGGDAGNGIKNGS

7990                ----AAGKNTFIDNINKVGK-KNPDGTVAPDAISENTVVNAKDLKNVVDT----------
CCM5995             ----HENKNTFMENVDTIGK-AN--------VISENTVVNAGDLKNLVDT----------
IPDH                ----HENKNTFMKNVNTIGD-TN--------GISENTVVNAGDLKNLVDT----------
10672-6             ----TSGKNTFIKKLEKVGG-TGE------GAIDKTTVVNAGDLKNLADT----------
CCM5974             TVDSTQGNNTFIKNLEKVGG-TDT------DAIDKTTVVNAGDLKNLADT----------
F149                ------------------------------------------------------------
4895                ----HENKNTFMENVDTIGK-AN--------GISENTVVNAGDLKNLVDT----------
Avicor              TIDGTTGNNTFIENLDKVGKLANE------GGIDKTTVVNAGDLKNLADTPLFFSGDSAD
CCM5976             TIDGTTGNNTFIENLDKVGKPANE------GGIDKTTVVNAGDLKNLADTPLFFSGDSAD
RecGab_2312(aa1-91) TIDGTTGNNTFIENLDKVGKPANE------GGIDKTTVVNAGDLKNLADT----------
```

```
7990                  KTLQSPLFFEGDSADGVA-NENDSDKNTFARKLSQKTKIVGGVDLGL-KENATAEDRKKAIAD
CCM5995               KTLQSPLFFEGDSADGVA-NENDSDKNTFARKLSQKTKIVGGVDLGL-KENATAEDRKKAIAD
IPDH                  KTLQSPLFFEGDSADGVA-NENDSDKNTFARKLSQKTKIVGGVDLGL-KENATAEDRKKAIAE
10672-6               -----PLFFQGDVSSATG------TNNTFSRKLSEKVSIVGEVKDGLEREDATAEEKAEAIRA
CCM5974               -----PLFFSGDAADGKEYNGTKNTKNTFGRKLSEEAKIVGGV----------------TTPA
F149                  KTLQSPLFFEGDSADGVA-NENDSDKNTFARKLSQKTKIVGGVDLGL-KENATADERKKAIAE
4895                  KTLQSPLFFEGDSADGVA-NENGSDKNTFARKLSQKTKIVGGVDLGL-KENATADERKKAIAE
Avicor                KTLQSPLFFEGDSADGVA-NENGSDKNTFARKLSQKTKIVGGVDLGL-KENATADERKKAIAE
CCM5976               KTLQSPLFFEGDSADGVA-NENGSDKNTFARKLSQKTKIVGGVDLGL-KENATADERKKAIAE
RecGab_2312(aa92-338) -----PLFFSGDSADGVA-AADGTDKNTFSRKLSQETKIVGGVDLGL-QAGATAEARKKAIAD

7990                  KLTDGNIGVISDGSNTLTVKLAKNLVNLTSVETGSGDNKTKMTE-------------------
CCM5995               KLTDGNIGVISDGSNTLTVKLAKNLVNLTSVETGSGDNKTKMTKNGITTTVKDGDTFKVTTTT
IPDH                  KLSDNNIGVVSNGTDTLTIKLAKNLVNLTSVETGSGDNKTKMTENGITTTVKDGDTFKVTTTT
10672-6               KLSDGNIGVVSNGTDTLTIKLAKELQNLTSVTTGSGDNTTVMNQ-------------------
CCM5974               NLTDNNIGVVSNGTDTLTIKLAKELVNLTSVETGSGNNKTKMTENGITTTVKDGDTFKVTTTT
F149                  KLTDHNIGVISNGTDTLTIKLAKDLTDLHSVTTTD----------------------------
4895                  KLTDHNIGVISNGTDTLTIKLAKDLTDLHSVTTTD----------------------------
Avicor                KLTDHNIGVISNGTDTLTIKLAKDLTDLHSVTTTD----------------------------
CCM5976               KLSDGNIGVISDGTDTLTIKLAKDLTDLHSVTTTD----------------------------
RecGab_2312(aa92-338) KLTNGNIGVISDGTDTLTIKLAKDLTGLNSSVYTS----------------------------

7990                  ---------------------------NGVTITTQTP--KNGDKPASTTETKLSKDGLATDG
CCM5995               PNGLATTEVKLDSEGMPKGESTTTMVTTDGVTITTQTP--KNGDKPASTTETKLSKDGLATDS
IPDH                  PNGLATTEVTLDSEGMPKGESTTTMVTTDGVTITTQTPPDPDGDKPASTTETKLSKDGLATDG
10672-6               ---------------------------DGVTITTQTP--KNGDKPASTTETKLSKDGLATDG
CCM5974               PNGLATTEVKLDSEGMPKGESTTTMVTTDGVTITTQTP--KNGDKPASTTETKLSKDGLATDG
F149                  ----------------------------------------DK---GNTTQINGKGLAT--
4895                  ----------------------------------------DK---GNTTQINGKGLAT--
Avicor                ----------------------------------------DK---GNTTQINGKGLAT--
CCM5976               ----------------------------------------DK---GNTTQINGKGLAT--
RecGab_2312(aa92-338) --------------------------------LTPAGNDGDA---TSSMKLDGNGIRF--
```

```
7990                   TIKV----TGDNAKDLVTIAKGTETDPNATDKEFGTVTIDGKNDSKATLTVDRGTKSVTDKAN
CCM5995                TVKVTNGKTGDDAKDLVTIAKGTETDPKATDKEFGTVTIDGKNDSKATLTVDRGTKSVTDKAN
IPDH                   TIKV----TGDNAKDLVTIGKG--TDGSSTDKEYGKIGLDGKNGSNATLTVDRGTKSVTDKAN
10672-6                TVKVTNGKTGDDAKDLVTIGKGTETDQNATDKEFGTVTIDGKNGSNATLTVDRGTKSVTDSAN
CCM5974                TVKVTNGKTGDDAKDLVTIGKGAKTDPNATDKEFGTVAIDGKNGSKATLTVDRGTKSVTDSAN
F149                   --------TADDGK--------------------GNIKI-------TTVTSDGITIVTTDK--
4895                   --------TADNGK--------------------GNIKI-------TTVTSDGITIVTTDK--
Avicor                 --------TADNGK--------------------GNIKI-------TTVTSDGITIVTTDK--
CCM5976                --------TADDGK--------------------GNIKI-------TTVTSDGITIVTTDK--
RecGab_2312(aa92-338)  --------------------------------------------------IDGQGKAKTDA--

7990                   ILKDPKQPAEDNNPVS-MDRLTYTTTGADGKSIIKHEVATLDDGFFLTTEHTKADETRTVLFN
CCM5995                ILKDPKQPAEDNNPVS-MDRLTYTTTGADGKSIIKHEVATLDDGFFLTTEHTEEDKTRTVLFN
IPDH                   ILKDPKQPAGDNNPVS-MDRLTYTTTGVGGKT-IEHQVATLDDGFFLTTEHTKADETRTVLFN
10672-6                VGKDPTNPIDKDNPVAGMDRLTYTTTGPNNET-INHEVATLDDGFFLTTEHTEANKTRTVLFN
CCM5974                VGKDPTKPIDKNNPVAGMDRLTYTTTGIGDKT-IEHQVATLDDGFFLTTEHTEADKTRTVLFN
F149                   ---------DGNNNVS------LTNKGLDN---GHHQIINVKSG-------------------
4895                   ---------DGNNSVS------LTNKGLDN---GHHQIINVKSG-------------------
Avicor                 ---------DGNNSVS------LTNKGLDN---GHHQIINVKSG-------------------
CCM5976                ---------DGNNSVS------LTNKGLDN---GHHQIINVKSG-------------------
RecGab_2312(aa92-338)  ----PS----------------LTTDGIKG---GNKQITNIGSG-------------------

7990                   HTIKVLDGANTKVSAMGGEKGTHTFSIDVTGLPVTYTATKTDANGAPTGEPVDVSKVGDGYQL
CCM5995                HTIKVLDGANTKVSAMGGEDGTHTFSIDVTGLPVTYTATKTDANGTPTGKPVDVSKVGDGYQL
IPDH                   HTIKVLDGANTKVSAMGGKDGTHTFSIDVTGLPVTYTATKTDTNGAPTGDSMDVSKVGDTYQL
10672-6                NTIKVLDGANTKVSAMGGKDGTHTFSIDVTGLPVTYTATKTDANGTPTGEPVDVSKVGDGYQL
CCM5974                NTIKVLDGANTKVSAMGGEKGTHTFSIDVTGLPVTYTATKTDANGAPTGEPVDVSKVGDGYQL
F149                   ---------------------------------------------------------------
4895                   ---------------------------------------------------------------
Avicor                 ---------------------------------------------------------------
CCM5976                ---------------------------------------------------------------
RecGab_2312(aa92-338)  ---------------------------------------------------------------

7990                   ADGTKLVKVDD-KYYKPEQLENNKPKPGEEGLTIKQSVSLVQDNGAPATLERVGSGRRNGMNT
CCM5995                ADGTKLVKVGD-KYYTPDQLENGKPKPNVEGLTIKESVSLTQDNSAPATLERVGSGRRNGMKA
IPDH                   ADGTKLEKVGD-KYYTPDQLENGQPKPNAEGLTIKQSVSLVQDNGAPATLERVGSGRRNGMNA
10672-6                ADGTKLVKAGDNKYYTHDQLDNGQPKPDAEGLTIKESVSLTQDNSAPATLERVGSGRRDGMKA
CCM5974                ADGTKLVKVDD-KYYKPEQLENNKPKPGEEGLTIKQSVSLVQDNGAPATLERVGSGRRNGMNA
F149                   ---------------------------------------LLDRQGKPVTLK----------NA
4895                   ---------------------------------------LRDRQGNPVTLK----------NA
Avicor                 ---------------------------------------LRDRQGNPVTLK----------NA
CCM5976                ---------------------------------------LLDRQGKPVTLK----------EA
RecGab_2312(aa92-338)  ---------------------------------------LRDRQGKPVTLK----------NA

7990                   KDT-PMDWNSILN-AAADNAEVNNMLTNAANIGDVRDAIKSITNGTDGNAIGGFGLTGNNGDV
CCM5995                DAD-PMVWDKILNPSAEDKAKVDNMLTNAANIGDVRDAIKSITDGTSDNASGGFGLTGNNGDV
IPDH                   KDT-PMDWNSILN-AAADNAEVNNMLTNAANIGDVRDAIKSITNGTDGNAIGGFGLTGNNGEV
10672-6                DAD-PMAWDKILNPSAADKAKVDNMLTNAANIGDVRDAIKSITDGTSDNASGGFGLTGNNGDV
CCM5974                NKDTPMDWNSILN-AAADNAEVNNMLTNAANIGDVRDAIKSITNGTDDNAIGGFGLTGNNGEV
F149                   SGD-------ILN--------------NAVNVKDLRDVVQGLTDSGKG---GGFGLTGNNGEV
4895                   SGD-------ILN--------------NAVNVKDLRDVVQGLTDSGKG---GGFGLTGNNGEV
Avicor                 SGD-------ILN--------------NAVNVKDLRDVVQGLTDSGKG---GGFGLTGNNGEV
CCM5976                SGD-------ILN--------------NAVNVKDLRDVVQGLTDSGKG---GGFGLTGNNGEV
RecGab_2312(aa92-338)  SED-------ILN--------------NAVNVRDLRDVVQGLTDSGKG---GGFGLTGNNGEV

7990                   KQDLGKTITVKGGIANKMDNAGTDLGQPAKSTTDKNTYVNVKDVPNADGKGSHKEMVI
CCM5995                KQDLGKTITVKGGIANKVDKAGQDLHVPAKSTTDKNTYVNVKDVPNADGKGSHKEMVI
IPDH                   KQDLGKTITVKGGIANKKDDSDKDLGQPAKSTTDKNTYVNVKDVPNADGKGSHKEMVI
10672-6                KQDLGKTITVKGGIANKVDEAGQDLHVPEKSTTDKNTYVNVKN----------NEMVV
CCM5974                KQDLGKTITIKGGIETKHD-AATNKDVPAKSTTDKNTYVNVKDVPNADGKGSHKEMVI
F149                   RKSLGETITLKGGIANKIE-NGIDMNKPEKATTDKNTYIDVKTSKN----GSEKIMVV
4895                   HKSLGETITLKGGIANKLE-NGIDMNKPEKATTDKNTYIDVKTSEN----GSEKIMVV
Avicor                 HKSLGETITLKGGIANKLE-NGIDMNKPEKATTDKNTYIDVKTSEN----GSEKIMVV
CCM5976                HKSLGETITLKGGIANKVE-NGRDMNKPEKATTDKNTYIDVKTSKN----GSEKIMVV
RecGab_2312(aa92-338)  HKSLGETITLKGGIANKIE-NGIDMNKPEKATTDKNTYIDVKTSKN----GSE-----
```
